# “You’re an expert in me”: the role of the generalist doctor in the management of patients with multimorbidity

**DOI:** 10.15256/joc.2015.5.65

**Published:** 2015-11-20

**Authors:** David Haslam

**Affiliations:** ^1^National Institute for Health and Care Excellence, London, UK

**Keywords:** Comorbidity, multimorbidity, multiple chronic conditions, primary care, general practice, guidelines, NICE, polypharmacy, generalist doctor

It is not often that a single patient successfully symbolizes almost every important trend in modern medicine, but one particular man achieved this in a solitary consultation. He was a patient in my general practice. He was 77 years old – exemplifying the ageing population, and had only lived in my area for 3 or 4 years – exemplifying an increasingly mobile population. I knew him well and saw him often – combining an aspiration for continuity, and increasing consultation rates in primary care. He had prostate cancer, but he also had hypertension, diabetes, coronary artery disease, macular degeneration, hyperlipidaemia, an arthritic right hip, and, hardly surprisingly, depression. This was multimorbidity par excellence.

He took 17 tablets a day, which is not unusual these days for someone with hypertension and diabetes – exemplifying the challenge of polypharmacy, and on this particular occasion, he had written to me before the consultation enclosing copies of a paper from the *Lancet* and another from the *New England Journal of Medicine*, both on the topic of the possible management of his prostate cancer – which absolutely exemplifies the changes brought about by the digital world, increasing availability of information for patients, and a major and positive change in the doctor–patient relationship.

In his letter, he suggested that I might like to read the papers before our next consultation, as he would like to discuss them with me, which for me absolutely exemplified the challenge of the time constraints on busy doctors and other clinical staff. 

When he came in, I thanked him for his letter and the papers, but I stressed to him that I am no expert in prostate cancer and that I felt that he really needed to discuss the papers with his urologist. I will never forget his response. “I know you’re not an expert in prostate cancer”, he said. “You’re an expert in me”. And he was right, and very wise. He understood the key role of the generalist doctor in the world of multimorbidity, and this really difficult balance we have to make between the ever-increasing levels of detailed scientific knowledge that can help our patients, and the need to understand and treat the whole person, the whole patient.

I have often thought that focusing on a single condition is like having a few beautifully clear and focused pixels, without having the whole picture. And without the whole picture, who knows what we are really looking at and treating?

Multimorbidity is very much the key focus of this journal, and the challenges that it brings have been well described. In a healthcare system designed around single conditions, with hospital departments of cardiology, respiratory medicine, gastroenterology, and the rest, comorbidities can feel like a nuisance, rather than the norm. But as Stewart Mercer and others have shown [1], there are more people in the UK with two or more long-term conditions than there are with one long-term condition. And this is not just a matter of ageing. There are more people with two or more comorbidities under the age of 65 years than there are over the age of 65 [2]. So it is unimorbidity that is the outlier, the less usual state of affairs, and comorbidity and multimorbidity, which are the norm.

The impact is not just on the organization of care. Much research excludes patients with comorbidities, producing detailed and accurate research on potentially highly unusual populations. Guidelines too become a challenge. Within the National Institute for Health and Care Excellence (NICE), as one of the world’s foremost producers of guidelines, we absolutely recognize that simply producing more and more guidelines based on single conditions is not the whole answer, and we are working on guidelines on the management of multimorbidity to be published in the autumn of 2016.

Indeed, unthinking application of single-condition guidelines carries a very real risk of triggering over-treatment and polypharmacy. We know that in England, the average elderly person in a care home will be on nine different medications [3]. Nine is the average, but for many it is even more. The evidence base for the benefits of such polypharmacy is poor, and the risk of side effects is surely magnified with every drug that is added, sometimes because the prescriber feels that the guidelines tell them that they have to do this.

In fact, every piece of NICE guidance carries the following extraordinarily important words: “*Healthcare professionals are expected to take it fully into account when exercising their clinical judgement. However, the guidance does not override the individual responsibility of healthcare professionals to make decisions appropriate to the circumstances of the **individual patient**, **in consultation with the patient** and/or guardian or carer, and informed by the summaries of product characteristics of any drugs*”.

These are vitally important words, but raise the question as to how many healthcare professionals read them? It would be a concern if people treat these like the terms and conditions they face when downloading new software. The number of doctors who complain to me that NICE guidelines do not allow for patient autonomy and the complexity of multimorbidity shows that they hurry past these important words to get to the “real facts”… the dose of the drug, the diagnostic test, etc. Within NICE, we are certainly trying to address the human factors that get in the way of people taking this message firmly on board. Understanding this issue really is critically important.

Indeed, the complexity of multimorbidity flags up another major issue. If you have eight different long-term conditions (and I well remember a patient with coronary artery disease, hypertension, diabetes, hyperlipidaemia, chronic kidney disease, macular degeneration, osteoarthritis of the hip, and depression) – then, what does good care look like? It is unlikely that it will look like a simple addition of the guidelines on all eight conditions. Indeed, it seems clear that the only person who can say what appears to be good for a person with these comorbidities is the patient themselves.

And, in addition, we are absolutely stressing how incredibly important it is to work with patients – understanding their needs and aspirations, and, to coin a phrase, “doing medicine with patients rather than doing it to them”. Taking into account the needs and aspirations of the individual patients and their families should absolutely move healthcare away from the many aspects of frankly ludicrous polypharmacy. The mother of a colleague of mine suffers from very severe dementia, and has had a recent major stroke, and a fractured hip. Whilst in hospital, she was started on a statin. When challenged as to the appropriateness of this therapy, the prescribing doctor said, “I prescribed it because that’s what the guidelines say I should do.” 

What the guidelines said was that the prescriber should take into account the circumstances of the individual patient. Simply adding therapies because of a misreading of the function of guidelines is not good medicine. It is thoughtless medicine. And prescribing is an activity that justifies considerable thought.

Polypharmacy as a side-effect feature of multimorbidity is an issue that is of major importance. There is infinitely more research into the benefit of adding therapies than there is into the benefit of stopping treatments, but with vast numbers of patients taking multiple drug regimens with poor evidence of how the various therapies might interact, is an area that needs far greater attention.

Indeed, NICE is giving this area a great deal of attention. Whilst NICE guidelines on managing multimorbidity will be published in September 2016, there has already been work into medicines optimization [4], and into the social care of older people with multiple long-term conditions [5].

But multimorbidity raises many more challenges than guidelines, research, and polypharmacy. The whole organization of secondary care medicine over the past 60 years has been based around single conditions or systems – with departments of cardiology, respiratory medicine, and such like. As multimorbidity becomes the norm, there is an ever-increasing need to train generalists – a development strongly recommended by the Future Hospital Commission in its report for the Royal College of Physicians [6].

And yet, consistently and bizarrely, many within medicine see generalism as an unattractive career choice. In the UK, USA, and in many other countries, the smaller a clinician’s area of expertise, the higher his or her prestige. The more one thinks about this logically, the more counterintuitive it is. Generalists are seen as being a lower form of medicine – unattractive to many students, or at best a career phase to be endured en route to a specialism. 

In an era of multimorbidity, generalist doctors become increasingly important. Without the availability of generalists in primary and secondary care, patients face having to see multiple specialists, a situation which is frequently a recipe for poor communication, poor holistic care, wasted patient time, and considerable duplication and waste. 

And, of course, the real “expert in me” is not the doctor. It is the patient. Recognizing that will be the real revolution.
